# Supplementary material for: Content-rich biological network constructed by mining PubMed abstracts
Source: BMC Bioinformatics. 2004 Oct 8;5:147. doi: 10.1186/1471-2105-5-147 (PMC528731; doi:10.1186/1471-2105-5-147)
Supplement: Additional File 2 — The original results of the above study (non-essential files are deleted to keep the file size under the limit set by BMC bioinformatics). [file 1471-2105-5-147-S2.bz2 › chilibotAdditionalFile2/dip10/601ID10052460E10980/html/HOXB1_PBX1.html]

 


 **HOXB1** and **PBX1** 
  
Found 178 abstracts in PubMed, retrieved 10.  
 

 What does Google say? 
 PDF only 
| .edu only 

---

**Interactive relationship** (e.g. stimulation, inhibition, etc)

**Neutral relationship**- This observation implies that this region is at least partially preformed and thus ready to interact with  **Pbx1**  and stabilize binding of  **Pbx1**  and  **HoxB1**  to DNA.  Ref: 11369863 Protein Sci, 2001
- The  **HoxB1**  hexapeptide is a prefolded domain implications for the  **Pbx1**  Hox interaction.  Ref: 11369863 Protein Sci, 2001
- Like PREP1, PREP2 PBX  [ **PBX1** ]  heterodimers are capable of forming ternary complexes with  **HOXB1** .  Ref: 11972344 Nucleic Acids Res, 2002
- Herein we describe NMR structural calculations of the hexapeptide of  **HoxB1**  Nalpha acetyl Thr Phe Asp Trp Met Lys amide that has been shown to mediate binding between  **HoxB1**  and  **Pbx1**  and a hexapeptide consensus sequence Nalpha acetyl Leu Phe Pro Trp Met Arg amide.  Ref: 11369863 Protein Sci, 2001
- The structures of the  **HoxB1**  peptide and the trans form of the consensus peptide reveal a turn very similar to that found as part of the  **HoxB1**   **Pbx1**  DNA complex in the X ray crystal structure.  Ref: 11369863 Protein Sci, 2001
- However, on the  **hoxb1**  autoregulatory enhancer, Hoxa1 and Hoxa1 NA KR may display distinct activity despite the presence of Pbx  [ **PBX1** ] , in a cell type dependent manner.  Ref: 12060683 Nucleic Acids Res, 2002

**Non-interactive relationship** (e.g. studied together, co-existance, homology, etc.)

- We previously showed that  **HOXB1**  and  **PBX1**  activate transcription from the b1 ARE by binding to sequences required for the expression of a reporter gene in rhombomere 4 in vivo.  Ref: 11278854 J Biol Chem, 2001
- We show that, in P19 embryonal carcinoma cells, GCs potentiate RA induced expression of the murine Hoxb 1  [ **HOXB1** ]  gene through an autoregulatory element, b1 ARE, recognized by the  **Pbx1**  and  **HOXB1**  homoeodomain proteins.  Ref: 12487626 Biochem J, 2003
- Therefore, we hypothesize that expression of two additional mesenchymally derived nuclear transcription factors, retinoid X receptor alpha RXR alpha and homeobox  [ **PBX1** ]  b 5 Hoxb  [ **HOXB1** ]  5, would also be altered in hypoplastic lungs.  Ref: 12505043 J Surg Res, 2002
- Analysis of two distinct retinoic acid response elements in the homeobox  [ **PBX1** ]  gene  **Hoxb1**  in transgenic mice.  Ref: 11891985 Dev Dyn, 2002
- Additional transfection experiments showed that the  **Pbx1**   **HOXB1**  heterodimer was the target for the GC effect.  Ref: 12487626 Biochem J, 2003
